# Spiritual nursing education programme for nursing students in Korea: a systematic review and meta-analysis

**DOI:** 10.1186/s12912-024-01961-6

**Published:** 2024-05-07

**Authors:** Hyun-Jin Cho, Kyoungrim Kang, Kyo-Yeon Park

**Affiliations:** 1 College of Nursing, Pusan National University, 49 Busandaehak-ro, Mulgeum-eup, Yangsan-si, Gyeongsangnam-do 50612 South Korea; 2 College of Nursing, Research Institute of Nursing Science, Pusan National University, 49 Busandaehak-ro, Mulgeum-eup, Yangsan-si, Gyeongsangnam-do 50612 South Korea

**Keywords:** Education, Meta-analysis, Spiritual nursing, Students, Systematic review

## Abstract

**Purpose:**

This study conducts a systematic review and meta-analysis to understand the characteristics and contents of studies on spiritual nursing education programmes and their effects.

**Methods:**

The literature search included five databases (RISS, KISS, DBpia, Science ON, and KmBase) published in South Korea until September 30, 2021. Nine studies were included in the final review, with six for the meta-analysis using the RevMan 5.4. 1 programme. The programmes targeted nursing students and nurses in the RN-BSN course and employed methods such as lecturing, discussions, and case presentations. The contents focused on self-spirituality awareness, spirituality-related concepts, understanding others’ spirituality, and the process and application of spiritual nursing.

**Results:**

The meta-analysis revealed statistically significant effects on spiritual nursing competencies, spirituality, spiritual well-being, existential well-being, and spiritual needs, except self-esteem. Spiritual nursing education was effective in enhancing spiritual nursing competencies.

**Conclusion:**

The study confirmed that spiritual nursing education effectively improves spiritual nursing competency, indicating a need for increased focus and administrative and financial support for such education in schools and hospitals. Furthermore, future studies should employ randomised experimental designs to examine the effects of online education programmes with short training time on clinical nurses in hospitals.

**Supplementary Information:**

The online version contains supplementary material available at 10.1186/s12912-024-01961-6.

## Introduction

Spiritual nursing involves providing care that recognises and responds to the spiritual needs of people in specific situations such as birth, trauma, illness, and loss [1]. It can provide answers to fundamental questions such as meaning of life, suffering, distress, and death through the person’s inner healing resources [2]. The physical, mental, social, and spiritual aspects of human beings dynamically interact with one another, with the spiritual aspect actively integrating and regulating all aspects [3]. The goal to enhance understanding and management of human health and disease has led to a growing interest in the spiritual aspect [4]. Spiritual nursing, as a trend, is reinforced as an essential obligation in modern nursing [5], which pursues holistic health management [6]. Therefore, spiritual nursing is considered an important core concept of holistic nursing [7].

Despite the importance of the spiritual aspect in health, most nurses have never received training in spiritual nursing and have little experience in utilizing it [8]. Nursing college students lack clinical practicum opportunities in spiritual nursing, hindering them to gain experience in this area [9]. Additionally, nurses often avoid spiritual nursing because they perceive it as a religious concept or as unscientific owing to the abstract nature of the spiritual aspect and their lack of knowledge [10]. Moreover, spiritual nursing can be challenging owing to lack of time, prioritisation of physical nursing tasks, and inadequate staff training [11, 12].

Spiritual nursing education has been proposed as a solution to these problems, leading to the development of spiritual nursing education programmes [11]. Nurses must learn about spirituality and spiritual nursing to enhance patients’ quality of life, health, well-being, coping mechanisms, and decision-making [13]. Spiritual nursing education serves as means to integrate spiritual aspects into comprehensive patient care [14]. Previous studies have shown that spiritual nursing education increases nurses’ ability to assess spiritual needs, enhances their competency in spiritual care, and improves their performance in spiritual nursing [11]. Moreover, nurses’ positive attitudes towards spiritual nursing influence their intention to engage in spiritual nursing and provide spiritual care [4]. Therefore, spiritual nursing education should aim to improve nurses’ attitude towards spiritual nursing and promote the application of spiritual nursing [15].

Spiritual nursing education programmes apply various curricula, content, delivery, and evaluation methods [16]. The educational content varies and may include the definition of spirituality or spiritual nursing, personal spiritual awareness, understanding of spiritual anguish, communication skills, comparative religious studies, and spiritual nursing ethics. Similarly, the educational methods employed comprise lectures, online education, simulation, role-plays, videos, group discussions, individual reflections, and practice [11, 13, 14]. This diversity arises from the lack of consensus on the meaning of spirituality and the unclear content and evaluation methods for spiritual nursing education programmes [1]. However, it also offers the opportunity to learn different approaches to caring for individuals from different social, cultural, and spiritual backgrounds [13]. Furthermore, diverse education programmes can serve as foundation for applying the most effective approach in situations requiring spiritual nursing education. Therefore, a systematic review can help in identifying strategies for integrating spirituality and spiritual nursing by examining the contents and teaching and evaluation methods of spiritual nursing education programmes.

Although several studies abroad conducted systematic reviews of spiritual nursing education programmes [11, 13, 14], only one phenomenological study [17] examined the experiences that Korean nursing students could potentially gain through spiritual nursing practicum and only one randomised controlled trial (RCT) study [18] investigated the effectiveness of spirituality training programmes focusing solely on spirituality on Korean nurses. Thus, a systematic review of spiritual nursing education programmes in Korea is necessary. In particular, Christianity began as a medical mission in Korea, establishing Korea’s first modern hospital, and subsequently expanded [19]. Consequently, nurses confused spiritual nursing with medical mission and perceived spiritual nursing as a compulsion to a certain religion [20], or Christian evangelism [21]. Moreover, the Korean culture values dignity and the views of others rather than the personal factors of self-satisfaction. The pursuit of spirituality also values harmony with the absolute, others, ancestors, and society [3]. Therefore, a spiritual nursing education programme that considers Korea’s cultural practices is necessary [22] to implement spiritual nursing in the nursing field.

Thus, using previous studies’ results related to the spiritual nursing education programmes in Korea, this study aims to systematically and scientifically integrate the contents, methods, and effects of these programmes. It seeks to develop and apply an effective programme for evidence-based practice, providing evidence for enhancing spiritual nursing competency and guiding future research directions.

## Methods

### Study design

This study is a systematic review that aims to understand the characteristics and effects of spiritual nursing education programmes on nurses and nursing students in Korea. The study used the systematic review manual of the National Evidence-based Healthcare Collaborating Agency [23]. The protocol for this review was registered in the International Prospective Register of Systematic Reviews (PROSPERO, ID: CRD42022326776).

### Search strategy and study selection

Studies published up to September 30, 2021, were examined using electronic databases. Earlier studies [10, 12, 13] published in 2015, 2016, and 2021 conducted systematic literature reviews of spiritual nursing education programmes using foreign databases and did not search the domestic literature suitable for the eligibility criteria of this study. Therefore, this study utilized the five most used databases in Korea: the Research Information Sharing Service (RISS), Korean Studies Information Service System (KISS), DataBase Periodical Information Academic (DBpia), Science ON (formerly National Discovery for Science Library [NDSL]), and KmBase. There was no restriction on the search period, and all documents corresponding to related subject words were searched until the search date (September 30, 2021). To increase sensitivity of the literature search, grey literature was manually searched using Google Scholar. Furthermore, additional literature was searched by reviewing the reference lists of studies obtained through the database search. ‘Nurse’, ‘nursing student’, ‘spiritual nursing’, ‘education’, and ‘programme’ were used as literature search terms. Three researchers independently performed the literature selection process. Intervention studies on the effectiveness of spiritual nursing education programmes for nurses or nursing students were included, while review articles, conference abstracts, or unpublished manuscripts were excluded. The full inclusion and exclusion criteria are presented in Table 1.

**Table 1:** Study inclusion and exclusion criteria

Category	Inclusion criteria	Exclusion criteria
Participants	- Nurses - Nursing college students	- Other participants such as physicians, social workers, counsellors or nurse’s aide.
Intervention/exposure	- Spiritual nursing education programmes	- Educational programme focuses only on individual spirituality
Comparison	- Existing educational methods or studies without any intervention	- None
Outcomes	- Studies presenting statistics before and after the experiment and the number of experimental groups	- None
Study Design	- Intervention studies on the effects of spiritual nursing education programmes - Quasi-experimental studies and randomized experimental design studies	- Non-experimental design studies such as qualitative studies, descriptive or correlation studies
Publication type	- Published manuscripts - Theses (if a thesis and an academic paper overlap, the academic paper choice)	- Conference abstracts or reports
Country	- Korea	- Other countries
Language	- Korean or English	- Other language

### Data extraction and quality assessment

Relevant data were extracted using a standardised data collection form, which included information on authors, publication year, research design, study subjects (number, grade), programme characteristics (training place, type, session/time/period/evaluation time, education methods, conceptual framework, and contents), measurement tools, variable measurement results, limitations, and suggestions (Tables 2 and 3). The Cochran’s Risk of Bias (Cochrane’s RoB 1) tool was used to evaluate the quality of the selected literature as randomised study [24]. Cochrane’s RoB 1 assesses seven areas: randomisation order generation, random assignment order concealment, blinding of study participants and researchers, blinding of outcome evaluation, insufficient data, selective reporting, and other biases. Each area was rated as having low, high, or uncertain risk of bias in the literature. Non-randomised studies were evaluated using the Risk of Bias Assessment Tool for Non-randomised Studies (RoBANS) [25] developed by the National Evidence-based Healthcare Collaborating Agency [23]. RoBANS assesses six areas: subject group selection, confounding variables, intervention (exposure) measurement, blinding outcome evaluation, incomplete data, and selective outcome reporting. Each area was evaluated as having low, high, or uncertain risk of bias. In this study, the risk of bias is considered low for non-randomised studies if the subjects were similar in the experimental and control groups and were prospectively and continuously recruited. The risk of bias is considered low also if the intervention was made after confirmation of exposure to spiritual nursing-related education, a questionnaire with confirmed reliability and validity was used, blinding to the outcome evaluator was reported, dropouts and reasons were reported or missing values did not affect the outcome, or the outcome value for the pre-defined outcome variable was reported. Three researchers independently evaluated each piece of literature, and any disagreements were resolved through discussion.

**Table 2:** Characteristics of included studies

Authors (year)	Study design	Sample (Exp./Cont.) Grade	Setting/ Regular or Special	Session/ Length/ Duration (F/U)	Teaching & evaluation methods (Materials)	Conceptual framework	Measure scales	Outcome measures			Limitations & suggestions
Kim et al. (1999)	One group pre-posttest design	SN 84 Junior	University/ Regular	14/ 120 min/ 14weeks	Lecture, Discussions, Presentation (PPT)	Not reported	Questionnaire focused on the attitudes toward death and dying	The dying patient’s emotional and physical needs Telling the truth of dying process	*p* = .352 *p* = .069		- Follow up survey - Subject to nurses
								Attitudes of medical personnels	*p* = .214		
								General attitudes on death and dying	*p* = .242		
								Attitudes toward mechanical assistance for life-expanding of helpless patient	*p* = .046		
								Attitudes of family members of dying patient	*p* = .621		
								Special facility and educational preparation for dying patient	*p* = .000		
								Special facility and welfare system for the old	*p* = .003		
Chung et al. (2011)	Nonequivalent Cont. pre-posttest design	SN 81 (42/39) Junior	University/ Special	12/ 60 min/ 6weeks	Lecture, MBTI test, Group discussions, Practice, Case presentation, Self-report (PPT, Videos)	Rogers’s person-centered theory ASSET model	SAS SWB SCCS	Spirituality Spiritual Well-being Spiritual Care Competency	*p* = .001 *p* = .004 *p* = .002		- No field training - Development of standard guidelines for spiritual nursing education
Choi (2014)	One group pre-posttest design	SN 83 Junior	University/ Regular	7/ 120 min/ 7weeks	Lecture, Case presentation, Role play (PPT, Videos)	Not reported	SWB SCCS	Spiritual Well-being - Spiritual Care Competency	*p* = .519 *p* < .001		- Regular curriculum
Hong (2016)	One group pre-posttest design	SN 65 Sophomore	University/ Special	16/ 60 min/ 16weeks	Lecture, Group discussions, Role play, Practice, Case study, Action learning, Self/team-report, Test (PPT)	ASSET model	SNS SWB SCCS	Spiritual Needs Spiritual Well-being Spiritual Care Competency	*p* < .001 *p* < .001 *p* < .001		- Only one group subject - No a comparison of the effectiveness of spiritual nursing education in various educational methods -A comparison of the effectiveness of spiritual nursing education in various educational methods - Subject to nurses
Jeong et al. (2016)	Nonequivalent Cont. non-synchronized with pre-posttest design	RN-BSN 93 (46/47) 1st & 2nd semester	University/ Regular	7/ 150 min/ 7weeks	Lecture, Case presentation, Case study (PPT, Videos)	ADDIE model Spiritual care module	SAS SNS SWB SCCS	Spirituality - Spiritual Needs Spiritual Well-being - Spiritual Care Competency	*p =* .095 *p =* .012 *p =* .121 *p* < .001		- Only BSN nurse - Subject to nurses - Extension of intervention period - Provide details and examples of intervention
Yoon et al. (2018)	Nonequivalent Cont. pre-posttest design	SN 60 (30/30) Senior	University/ Regular	8/ 120 min/ 8weeks	Lecture, Case presentation, Group discussions, Case study, Test (PPT, Videos)	Spiritual care module	SAS SNS SWB SCCS	Spirituality Spiritual Needs Spiritual Well-being Spiritual Care Competency	*p* = .001 *p* = .002 *p* = .016 *p* < .001		- Development of standardized intervention tools
Choi et al. (2019)	Nonequivalent Cont. non-synchronized with pre-posttest design	RN-BSN 63 (30/33) 1st & 2nd semester	University/ Special	7/ 90 min/ 7weeks	Lecture, Ego-gram test, Group discussions, Case presentation (PPT, Videos)	Not reported	RSE GICC EWB SCCS	Self-esteem - Communication - Existential Well-being Spiritual Care Competency	*p* = .365 *p* = .515 *p* = .025 *p* < .001		- Only BSN nurse - Subject to nurses - Short-term education for nurses at the hospital level
Kim et al. (2019)	Nonequivalent Cont. pre-posttest design	SN 80 (41/39) Senior	University/ Special	9/ 90 min/ 5weeks	Lecture, Ego-gram test, Group discussions, Case presentation, Self-report (PPT, Picture, Videos)	Psychological empowerment theory	RSE EWB TEQ SCCS	Self-esteem Existential Well-being Empathy Spiritual Care Competency	*p* < .001 *p* < .001 *p* < .001 *p* < .001		- Only two university nursing students - No follow up - Field training - Follow up survey - Develop standardized working protocols - Subject to nurses - Development of educational program and measurement tools for spiritual care in a pan-religious society
Lim et al. (2021)	Randomized controlled trials	SN 83 (42/41) Junior	University/ Special	10/ 120 min/ 5weeks (10weeks)	Lecture, Enneagram test, Group discussions, Case presentation, Relaxation, Self-report (PPT, Videos, Audio)	ASSET model	SAS EIS SWB SLC SCCS	Spirituality Ego-Identity Spiritual Well-being Satisfaction with Life Spiritual Care Competency	*p* < .001 *p* < .001 *p =* .050 *p* < .001 *p* < .001	*p* < .001 *p* < .001 *p* < .001 *p* < .001 *p* < .001	- Subject to senior student - Regular curriculum - Subject to nurses

Exp.=Experimental group; Cont.=Control group; F/U = Follow up; SN = Student Nurse; RN-BSN = Registered Nurse - Bachelor of Science in Nursing (The RN-BSN course allows a nurse who has completed an associate degree in nursing to obtain a bachelor of science in nursing); PPT = PowerPoint; MBTI = The Myers-Briggs Type Indicator; ASSET = a model for Actioning Spirituality and Spiritual Care Education and Training in nursing; ADDIE = Analysis, Design, Development, Implementation, Evaluation; SAS = Spiritual Assessment Scale; SWB = Spiritual Well-Being Scale; SCCS = Spiritual Care Competence Scale; SNS = Spiritual Need Scale; RSE = Rosenberg Self-Esteem Questionnaire; GICC = Global Interpersonal Communication Competence Scale; EWB = Existential Well-Being Scale; TEQ = Toronto Empathy Questionnaire; EIS = Ego-Identity Scale; SLC = Satisfaction with Life Scale

**Table 3:** Spiritual care education contents

Authors (year)	Self-awareness	Spirituality	Understanding others	Spiritual nursing process	Spiritual nursing application	Others
Kim et al. (1999)	-	6-7th Spiritual care concept, Preparation for spiritual care provider	-	8th Spiritual nursing process	9-14th: Spiritual nursing by situation	1st-5th: Death education
Chung et al. (2011)	1st: MBTI test 2nd: Holism	3rd: Spirituality, Spiritual healthy	4-5th: Stress management 6th: Giving hope 7th: Emotion 8th: Communication	9-10th: Assessment 11th: Diagnosis, Planning, Implementation, Evaluation	12th: Intervention practices	-
Choi (2014)	2nd: Self-assessment	1st: Spiritual care overview 2nd: Spiritual care preparation	5th: Communication	3rd-4th: Spiritual nursing practices (Bible, pray, music) 6th: Assessment, Diagnosis, Planning, Implementation	7th: Presentation of spiritual nursing cases.	-
Hong (2016)	2nd-3rd: Holism, Self-reflection	4–6th: Spirituality, Spiritual need, Spiritual well-being, Preparation for spiritual care provider	-	7-9th: Assessment, Diagnosis, Planning, Intervention, Implementation, Evaluation	10-14th: Spiritual nursing by situation: Role play 15-16th: Field practices	1st: Action learning understanding
Jeong et al. (2016)	1st: Holism, Self-world view	1st: Spiritual care necessity & history 2nd: Difference of spiritual nursing and general nursing 3rd: Spirituality, Spiritual healthy, Spiritual need, Spiritual difficulty 4th: Spiritual well-being, Preparation for spiritual care provider	-	5th: Assessment, Diagnosis 6th: Planning, Intervention, Evaluation	6th: Case study 7th: Case presentation	-
Yoon et al. (2018)	1st: Holism, 9th: Self-reflection	1st: Spiritual care necessity 2nd: Spiritual care history, Difference of spiritual nursing and general nursing 3rd: Spirit, Spirituality 4th: Spiritual need 5th: Spiritual well-being, Spiritual distress, Preparation for spiritual care provider	-	6th: Assessment, Diagnosis 7th: Planning, Intervention (Therapeutical oneself, Bible, Hymn, Prayer, Refer to priest), Evaluation	8th: Case presentation	9th: Exam
Choi et al. (2019)	2nd: Ego-gram test	1st: Spiritual care concept & necessity 3rd: Spirituality, Spiritual healthy	4th: Communication, Empathy	5th: Assessment, Diagnosis 6th: Intervention (Laughter therapy, Listening to music, Singing, Hope cycle, Meditation, Supporting religious Activities, Spiritual counseling)	7th: Case study presentation	-
Kim et al. (2019)	2nd: Ego-gram test	1st: Spiritual care define 3rd: Spirituality, Spiritual well-being, Spiritual distress	5th: Empathy 6th: Communication	7th: Assessment, Diagnosis 8th: Intervention (Support for religious activities, Spiritual counseling, Offer support group, Meditation, Giving hope, Laughter therapy, Music therapy, Biofeedback) 9th: Planning, Implementation, Evaluation	-	-
Lim et al. (2021)	1st: Enneagram test 2nd: Holism	3rd-6th: Spirituality, Spiritual healthy, relaxation	7th: Communication	8th: Assessment, Diagnosis 9th: Planning, Implementation, Evaluation	10th: Case presentation	-

### Data synthesis and meta-analysis

For studies where quantitative synthesis was possible, we conducted a meta-analysis using the RevMan 5.4.1 programme from the Cochrane Library. This was performed when the same outcome variables could be analysed, or when pre- and post-mean and standard deviation values for the outcome variables were available. Subgroup meta-analysis was performed when at least two studies had the same outcome variables. In calculating the effect size, the result variables of each synthesised study were analysed as continuous variables, with mean and standard deviation. The Standardised Mean Difference (SMD) was selected as the analysis method for effect size of the same outcome variable. The statistical significance level for effect size was set at 0.05, and the confidence interval was set at 95%. Heterogeneity between studies was assessed for the common part in the confidence interval and effect estimate using a meta-analysis forest plot, a visual method. Heterogeneity was quantitatively evaluated using Cochrane’s chi-square test and Higgins’ I^2^ statistic value. The I^2^ value is 0% when there is no heterogeneity, 30-60% when there is moderate heterogeneity, and more than 75% when there is large heterogeneity [23].

Analysis was conducted using a random-effects model, which adjusts weights to account for intersubject variation and heterogeneity between the studies used in meta-analysis. Given the diversity in samples, intervention methods, intervention period, and measurement tools across studies, the random-effects model was used when the heterogeneity was I^2^ = 50% or higher. When inputting data, if the outcome variables were measured twice, only the value calculated immediately after training was included. If the standard deviation for the difference before and after education was missing, the correlation coefficient calculated in another study was used and the missing standard deviation was replaced using the correlation coefficient [23].

## Results

### Study selection and characteristics

**Fig. 1:**
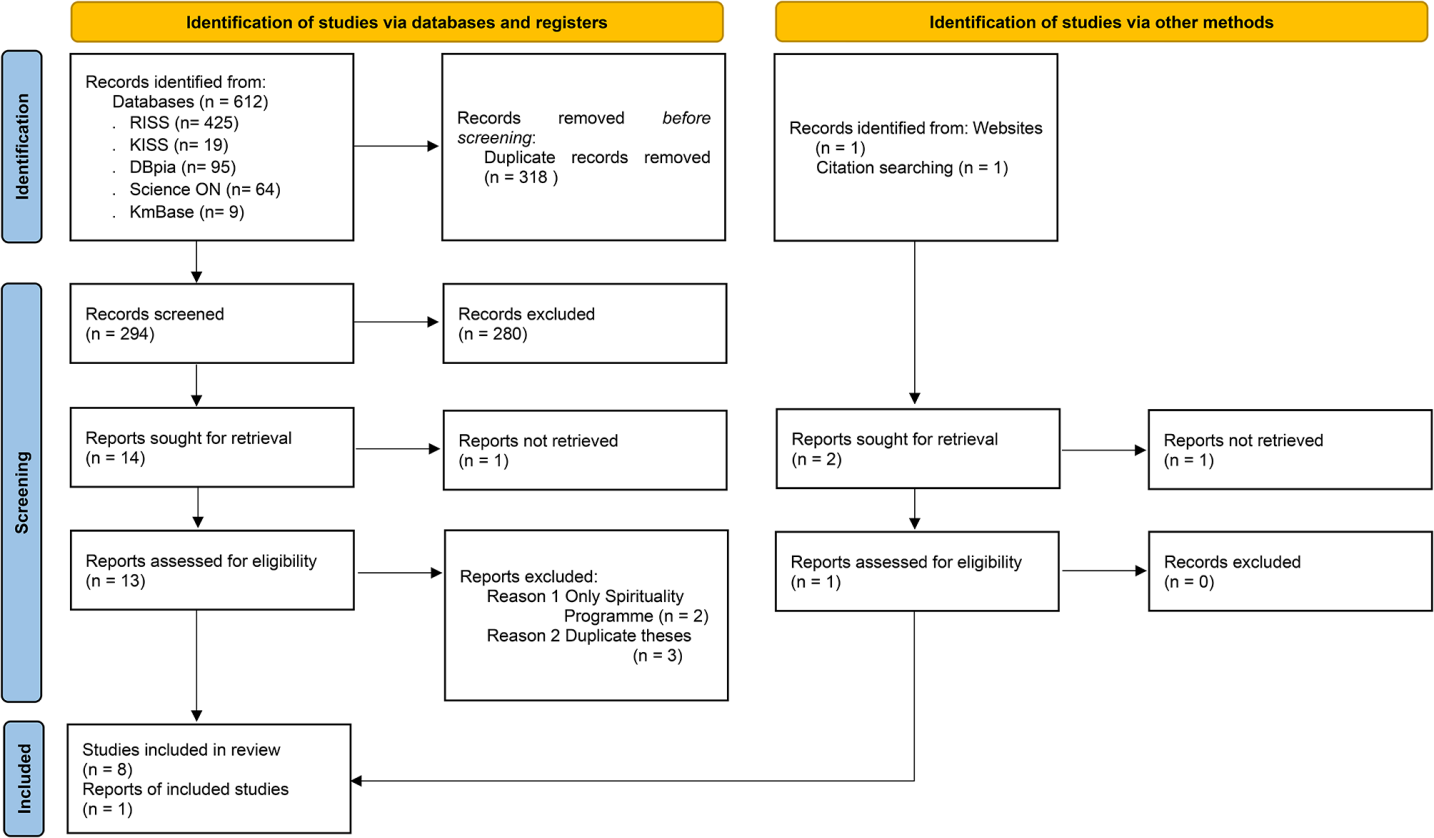
Flow diagram of study selection process

Literature was obtained using electronic databases, and a literature review was conducted according to the reporting guidelines recommended by the Preferred Reporting Items for Systematic Reviews and Meta-Analyses (PRISMA). We then carried out a literature search and identified 612 studies: 425 from RISS, 19 from KISS, 95 from DBpia, 64 from Science ON, and nine from KmBase. After removing 318 duplicate papers using RefWorks, we reviewed the titles and abstracts of 294 papers based on the inclusion and exclusion criteria; 280 studies that did not fit the study purpose were excluded. After a detailed review of the full texts of the remaining 14 studies in detail, one study with unverifiable original text, two studies focusing solely on spirituality rather than spiritual nursing, and three theses duplicating academic papers were excluded. Eight studies were thus included. Additionally, two studies found in the reference lists of the selected studies were reviewed, with one meeting the inclusion criteria. The original text of the other study could not be confirmed. Finally, nine articles were selected for the systematic review (see Additional file 1). Among these, six studies suitable for quantitative synthesis were meta-analysed. Disagreements among the three researchers were resolved through discussion. The exclusion of literature at each selection stages was recorded, and the document selection process was described using the systematic review flow chart [26] from the Preferred Reporting Items for Systematic Reviews and Meta Analysis 2020 (Fig. 1).

### Characteristics of selected literature

All nine studies considered were published in journals in Korea. Two studies each were published in 2016 and 2019, and one study each was published in 1999, 2011, 2014, 2018, and 2021. The study designs were as follows: three were single-group pre-post-quasi experimental studies, three were quasi-experimental studies with non-equivalent control groups, two were pre- and post-non-synchronised quasi-experimental studies with non-equivalent control groups, and one was a randomised control pre- and post-experimental study. Seven studies focused on nursing students, while two targeted nurses in a bachelor’s degree programme (Registered Nurse-Bachelor of Science in Nursing, RN-BSN). Among the nine studies, six included 80 to 99 participants, and three included 60 to 79 participants. Four studies targeted junior students, two targeted senior students, two targeted students in first and second semesters of the RN-BSN course, and one targeted sophomore students. All training sessions were conducted in university classrooms. Five programmes utilised non-formal education, while four were operated through regular education courses. The training session durations were as follows: four studies conducted training sessions for 120 min each, two studies for 60 min each, two studies for 90 min each, and one for 150 min. As for the number of sessions, three studies have had seven sessions each, while the remaining six studies have had eight, nine, 10, 12, 14, and 16 sessions, respectively.

### Characteristics of spiritual nursing education programmes

1) Teaching methods and content of spiritual nursing education programmes.

All nine studies in the review conducted lectures as a teaching method. Eight studies incorporated presentations, seven used discussions, three employed case studies, two featured practice sessions, and two included role-play. Additionally, relaxation techniques, action learning, and various tests were used. As for evaluation methods, four studies used reports and two used paper-written exams. Among the nine studies that provided lectures using PowerPoint (PPT) as an educational medium, seven also used various videos. Furthermore, one study presented photos, and another utilized sound equipment for relaxation. Six studies employed the conceptual framework, with three utilizing the Actioning Spirituality and Spiritual Care Education and Training in nursing (ASSET) model, two employing the textbook ‘Spiritual Nursing Module: Completion of Holistic Care’, one using the Analysis, Design, Development, Implementation, Evaluation (ADDIE) model, one applying the Psychological Empowerment Theory, and one implementing the Rogers Human-Centred Theory. Three studies did not mention any conceptual framework.

The content of spiritual nursing education was categorised into self-awareness, concepts related to spirituality, understanding of others, spiritual nursing process, and spiritual nursing applications. As for self-awareness, eight studies focused on examining the ego state to revise self-image through positive objective self-recognition in the first and second sessions of the programme. Each study included self-reflection in the third and ninth sessions. Four studies used tests such as ego-gram, MBTI, and enneagram tests, while five applied a holistic understanding of humans. The spiritual nursing process (assessment, diagnosis, planning, intervention, and evaluation) was included in all nine studies. Spiritual nursing intervention methods included therapeutic self-use such as being present together, attentive listening, touch, massage, lettering, poetry, laughter therapy, music therapy, occupational therapy, horticultural therapy, walking, meditation, use of the Bible, prayer, hymns using, support for religious activities, spiritual counselling, clergy referral, and offering support groups. The spiritual nursing process was covered in sessions 3 to 11 of the programme for training in spiritual nursing practice.

Eight studies applied spiritual nursing in various contexts: they targeted general, surgical, cancer, elderly, paediatric, and end-of-life patients, employed spiritual nursing in clinical situations, and explored spiritual nursing case studies and presentations. One study conducted a practicum at facilities for single mothers, the disabled, orphanages, and hospitals. Another study focused on nurses in bachelor’s degree programmes, asking them to apply their spiritual nursing education in practice by selecting patients themselves. The application of spiritual nursing was typically introduced in the latter part of the programme for practical use in clinical settings. Additionally, some studies included Nightingale’s nursing philosophy as educational content, while others explored the meaning of life and death.

2) Effects of spiritual nursing education programmes.

The effectiveness of spiritual nursing education programmes was confirmed using measures such as spiritual care competency (eight studies), spiritual well-being (six studies), spirituality (four studies), spiritual needs (three studies), existential well-being (two studies), self-esteem (two studies), self-identity (one study), life satisfaction (one study), empathy (one study), communication ability (one study), and attitude towards death (one study). All surveys were conducted using self-report questionnaires. Measurements were taken before and after training in eight studies, and before, after, and five weeks after training in one study.

In eight studies that measured spiritual care competency as a major variable, all spiritual care competency levels showed significant increase after intervention in the spiritual nursing education programme. However, for the sub-domains of spiritual care competency, one study did not demonstrate any significant difference in ‘professionalisation and improving the quality of spiritual care’ and ‘referral to professionals’, while another study did not show significant difference in ‘communication’. Three studies did not specify subdomain, and some studies measured only the ‘assessment and implementation of spiritual care’, ‘professionalisation and improving the quality of spiritual care’, and ‘personal support and patient counselling’ among the elements of spiritual care competency.

Among the six studies focusing on spiritual well-being, four showed a significant increase in spiritual well-being, one found no significant difference in religious well-being in the subdomain, and another showed no significant difference in existential well-being. Regarding the four studies that measured spirituality as a major variable, three showed a significant increase in the degree of spirituality, while one showed no significant change in transcendence in the subdomain. In studies measuring spiritual need as the main variable, all three studies showed significant differences, although one reported a significant decrease in spiritual need. Two studies reported a significant increase in spiritual need, with one showing no significant difference in the subdomains of ‘love and peace’ and ‘the meaning and purposes of life’.

Both studies that measured existential well-being as the main variable showed a significant increase in its degree. However, only one of the two studies measuring self-esteem as a major variable showed a significant increase in the degree of self-esteem. The studies measuring self-identity, life satisfaction, and empathy as the main variables showed significant increases, while one study measuring communication ability showed no significant difference. One study measured attitude towards death as a major variable. A significant increase was found in the need for prolonging the life of terminally ill patients and for an organisation dedicated to protection facilities, dedicated personnel, and the elderly problem. However, no significant differences were found in response to dying patient’s needs, death notice, attitude towards the dying, general attitude towards death, and dying patient’s family problems before and after education.

### Evaluation of the quality of included literature

The quality evaluation of a randomised study using Cochrane’s RoB tool showed a low risk of bias. Random numbers were assigned using a random numbering programme to ‘generate random assignment order’, and the allocation table was covered in an opaque envelope to ‘hide allocation order’. For the items ‘blind for research participants and researcher’ and ‘blind for outcome evaluation’, the assignment table was managed by an assistant involved in the curriculum, thus preventing exposure of the order to the experimental and control groups until the start of spiritual education. As for ‘insufficient result data’, dropouts occurred in both groups for similar causes, and the risk of bias was rated as low in ‘selective reporting’ and ‘other bias’.

**Fig. 2:**
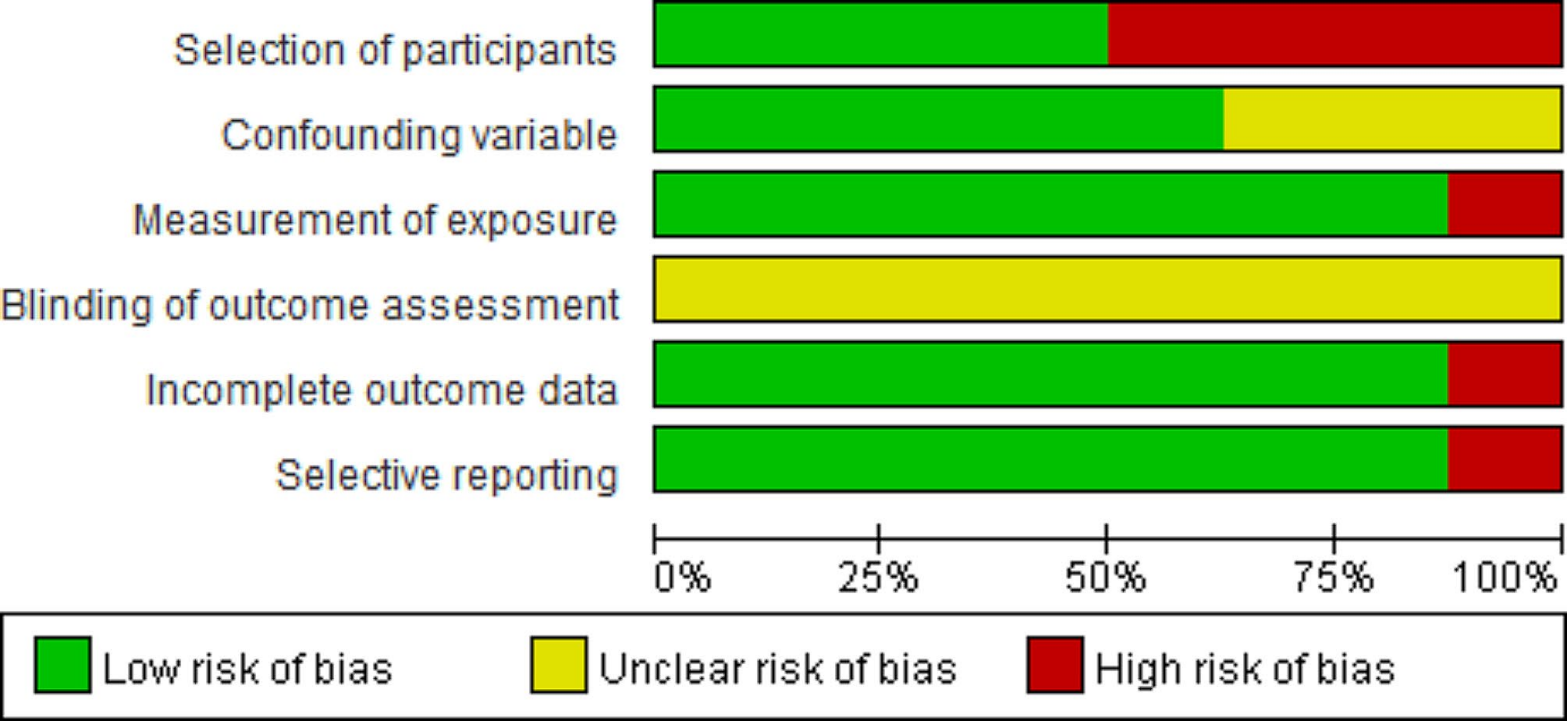
Risk of bias graph for non-randomized controlled studies

Of the eight non-randomised studies evaluated using the RoBANS tool, for the ‘subject group selection’ item, four studies confirmed that the experimental and control groups were the same or from the same period, and as the subjects did not receive spiritual nursing education at the time of study participation, it resulted in a low risk of bias. However, in one study, it was unclear whether there was an intervention in the study participants during the study participation. Additionally, in three studies, participants were not continuously recruited, leading to a high risk of bias. In terms of the ‘confounder variable’ items, five studies identified and appropriately considered the confounding variables, resulting in a low risk of bias, but the risk was uncertain in three studies. All eight studies used self-response for the ‘intervention exposure measurement’ item. Seven studies used the tools verified in previous studies, suggesting low risk of bias due to tool reliability. One study developed a tool by extracting from the literature, but as its validity and reliability were not presented, the risk of bias was high. The information reported in all eight studies was insufficient for the item ‘blindness for outcome evaluation’. ‘Incomplete outcome data’ showed low risk of bias in seven studies, except for one study with large difference in missing values between the groups. One study was evaluated as having high risk of bias for the ‘selective outcome report’ item owing to undefined results, while seven studies had low risk of bias by including all expected results (Fig. 2).

### Outcome variables and effect size of the included literature

**Fig. 3:**
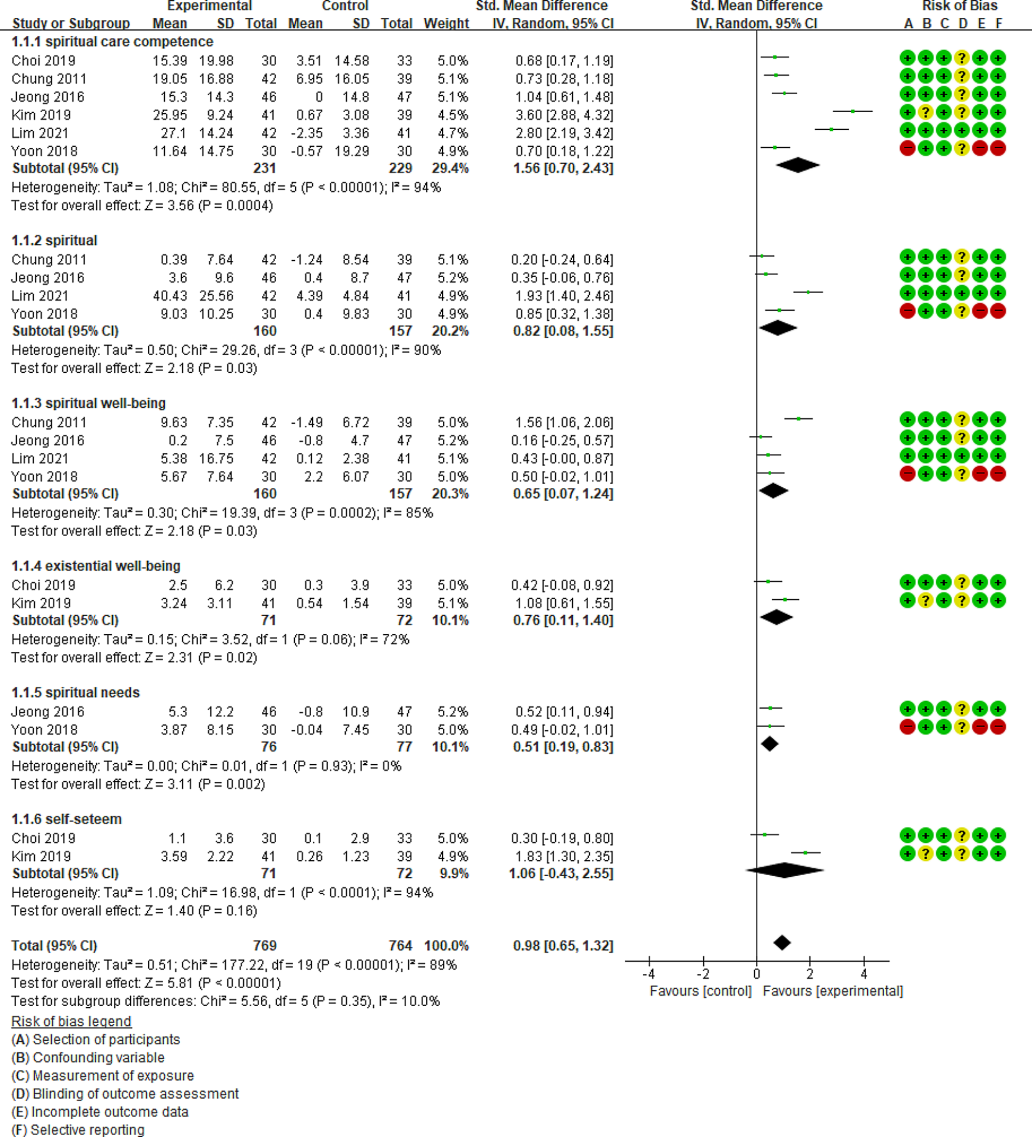
Forest plot of meta analysis on effects of spiritual care education

Of the nine studies, six compared the effects of a spiritual nursing education programme that included the control group that did not receive treatment. These studies presented the pre- and post-mean and standard deviation of the outcome variables, enabling effect size analysis (Fig. 3).

The examination of six studies measuring spiritual care competency as a major variable showed no homogeneity in the homogeneity test (Higgins I²=94%). As for effect size between the experimental and control groups of the programmes, the experimental group exhibited a 1.56 increase in spiritual care competency compared to the control group (*n* = 460, SMD = 1.56, 95% CI 0.70 to 2.43), indicating a statistically significant difference (Z = 3.56, *p* = .0004).

The examination of four studies measuring spirituality as a major variable showed no homogeneity in the homogeneity test (Higgins I²=90%). As for effect size, the experimental group showed a 0.82 increase in spirituality compared to the control group (*n* = 317, SMD = 0.82, 95% CI 0.08 to 1.55), indicating a statistically significant difference (Z = 2.18, *p* = .03).

The examination of four papers measuring spiritual well-being as a major variable showed no homogeneity in the homogeneity test (Higgins I²=85%). As for effect size, the experimental group exhibited a 0.65 increase in spiritual well-being compared to the control group (*n* = 317, SMD = 0.65, 95% CI 0.07 to 1.24), indicating a statistically significant difference (Z = 2.18, *p* = .03).

The examination of two studies measuring existential well-being as a major variable showed no homogeneity in the homogeneity tests (Higgins I²=72%). As for effect size, the experimental group showed a 0.76 increase in existential well-being compared to the control group (*n* = 143, SMD = 0.76, 95% CI 0.11 to 1.40), indicating a statistically significant difference (Z = 2.31, *p* = .02).

The examination of two studies measuring spiritual needs as a major variable showed no homogeneity in the homogeneity tests (Higgins I²=0%). As for effect size, the experimental group exhibited a 0.51 increase in spiritual need compared to the control group (*n* = 153, SMD = 0.51, 95% CI 0.19 to 0.83), indicating a statistically significant difference (Z = 3.11, *p* = .002).

The examination of two studies measuring self-esteem as a major variable showed no homogeneity in the homogeneity tests (Higgins I²=94%). As for effect size, the experimental group showed a 1.06 increase in self-esteem compared to the control group (*n* = 143, SMD = 1.06, 95% CI − 0.43 to 2.55), indicating no statistically significant difference (Z = 1.40, *p* = .16).

## Discussion

The systematic review and meta-analysis aimed to confirm the characteristics and contents of spiritual nursing education programme research, identify the effect of the spiritual nursing education programme, develop a spiritual nursing education programme by integrating education content and methods, and provide evidence for interventions strengthening the spiritual care competency.

This study confirmed that although interest and attempts in interventional research in spiritual nursing education are increasing, research on spiritual nursing education programmes in Korea is still not actively conducted. These results are similar to those of previous systematic reviews in other countries that showed increasing focus on spiritual nursing education for healthcare professionals [11, 14]. However, compared to the increase in observational studies on spiritual nursing [22], intervention studies on spiritual nursing education were insufficient and required comprehensive implementation.

As for the study participants, seven studies were conducted on nursing students, including those in the RN-BSN course. Nursing students are easier to recruit than nurses, as education for students is implemented in a classroom setting, eliminating the need for an intervention environment. However, in a systematic review of spiritual nursing education, Jones and Paal [11] identified 13 studies on nursing students and 14 studies on nurses conducted over the past ten years. Unlike in Korea, researchers in other countries actively conduct studies on intervention in educational programmes targeting nurses. Six studies included in this review also suggested that research on clinical nurses be conducted [6, 8, 10, 27–29]. It is crucial to explore whether spiritual nursing education programmes for nurses can affect the provision of spiritual nursing intervention to patients. Therefore, developing and actively applying practical spiritual nursing education programmes at the hospital level is necessary to enable the practice of spiritual nursing.

In terms of study design, eight out of nine studies were quasi-experimental ones, with three [27, 28, 30] among them using a one-group pre-post design. Difficulties could arise in designing a randomised controlled experimental study because students could not choose a class in regular course education, and time and psychological constraints pose challenges for students in special lecture-type education [31]. Limited availability of randomised controlled experimental studies led to the inclusion of quasi-experimental studies, which is a limitation of this study. Future studies should consider an RCT design to prevent subject selection bias and to clearly measure the effect of spiritual nursing education. Additionally, only one study performed a follow-up evaluation after five weeks of education [29], suggesting the need to consider a follow-up period to confirm continuity of the study results.

The duration of training sessions ranged from 60 to 150 min, with 120 min being the most common. This duration was likely based on the form of regular education or special lectures for nursing students and organised according to the average credits per class for bachelor’s degrees. Previous systematic review studies conducted abroad reported varying teaching hours, ranging from 30-minute to an all-day lectures [11]. This may be because, as found in a study on nurses, training was provided to them during lunch breaks or in the form of workshops, depending on the situation [32]. Previous studies have reported that spiritual nursing education was effective even in a short curriculum [11]. Therefore, when planning future research for three-shift nurses, it is necessary to consider short training duration to secure sufficient time for education.

In most studies, educational programmes were conducted in the form of general lectures, which is the most common educational method. However, research on nurses must address the shortcomings of face-to-face education considering the difficulty in adjusting nurses’ schedules owing to shift work. As previous international studies have proven the effectiveness of online spiritual nursing education [33–36], it is necessary to consider online non-face-to-face education. Additionally, field practice for spiritual nursing application was conducted in only one study [28], while two studies suggested research to combine theory and practice [10, 37]. As field practice prepare nurses to function in real situations [38], more studies should conduct field practices. Furthermore, for research targeting nursing students, considering that practicum is currently limited due to COVID-19, education through simulation must be applied as it is effective as actual clinical practicum in increasing confidence in nursing performance [39].

Regarding the content of spiritual nursing education, most programmes were based on the ASSET model, consisting of self-awareness, spirituality, understanding of others, and the spiritual nursing process and application. Most studies approached spirituality from a cross-religious perspective, with spiritual nursing intervention providing education focused on existential and religious well-being. This result is consistent with previous systematic review studies [11], where spirituality was taught not as part of religion, but comprehensively. Many people in Korea believe that spiritual nursing is related to a specific religion [20]. However, since Korea has various religions such as shamanism, Buddhism, Confucianism, and Christianity [3], spirituality was approached from various perspectives. Moreover, most of the spiritual nursing education programmes in Korea showed a holistic rather than religious approach [8]. This trend is also reflected in the definition of the multidimensional domain of spirituality and spiritual nursing adopted by the European Education Project for the Development of Standards for Spiritual Nursing Education [1]. Therefore, future studies should approach spirituality and spiritual nursing as multidimensional educational content meant to provide meaning and purpose to life beyond religion.

Studies in Korea focused on teaching communication skills to broaden the understanding of the spiritual needs of others and training on enhancing empathy and providing hope. The effectiveness of spiritual nursing education was measured by its relationship with the subject as a spiritual nursing provider, including communication, empathy, spiritual needs, and spiritual care competency. In contrast, previous international systematic reviews [11, 13, 14] focused more on recognising one’s own spirituality, training on self-reflection to broaden the understanding of individual spirituality, and measuring the changes in viewpoint, knowledge, and attitude towards individual spirituality and spiritual nursing. This difference may be attributed to the socio-cultural characteristics of Korea, which value harmony with family and community rather than individuals [3]. Thus, the educational content on spirituality and spiritual nursing in Korea focuses more on understanding others and performing spiritual nursing for the spiritual well-being of patients rather than on self-awareness or personal spirituality. Particularly, therapeutic communication skills are required to broaden the understanding of the spiritual needs of others, assess their spiritual needs, and support patients with a professional competency that improves spiritual nursing quality [8]. Therefore, a spiritual nursing education applicable to nursing practice, such as scenarios related to therapeutic communication and communication skills for certain situations, is necessary [8].

This systematic review found that spiritual nursing education programme improves spiritual care competency. This finding is consistent with the results of previous systematic reviews from other countries [11, 13, 14] and confirms the need for spiritual nursing education to address the challenges faced in related practice. All eight studies in this review used tools developed by Van Leeuwen and Tiesinga [40], but one study did not show any significant difference in ‘professionalisation and improving the quality of spiritual care’ and ‘referral to professionals’ from among the subfields of spiritual care competency [37]. Multidisciplinary collaboration between nurses and hospital clergy is necessary for a religious approach to the transcendental relationship of spirituality in performing spiritual nursing. As professional referrals are an important area of spiritual care competency that addresses the spiritual needs of the subject beyond the role of a nurse [40], a specific multidisciplinary approach in spiritual nursing education is necessary.

Studies measuring the effect of spiritual nursing education on spiritual well-being and spirituality showed different results, especially when checking the subdomains. No significant differences were found between the ‘religious well-being’ subdomains of spiritual well-being and the ‘transcendence’ subdomains of spirituality. This could be due to difficulties in reflecting the level of spirituality and spiritual well-being of subjects with no religion when measuring the ‘religious well-being’ subdomain of spiritual well-being or in the ‘transcendence’ subdomain of spirituality. Therefore, studies should revise and supplement the tools for measuring spirituality and spiritual well-being by reflecting the meaning of the changing concepts.

Some studies examined the effects of spiritual nursing education on spiritual needs. One study reported a decrease in spiritual needs after implementation of spiritual nursing education, suggesting ways to meet the spiritual needs of nurses through self-awareness and application of learnings [28]. However, two studies reported that increase in spiritual needs can be considered as increased sensitivity to one’s spiritual needs through spiritual nursing education and would help understand the spiritual needs of patients [6, 12]. These contradictory findings indicate that the reliability and validity of the measurement tool should be checked to confirm the effectiveness of spiritual nursing education and that presenting clear standards is necessary for result interpretation.

Studies in Korea used eleven variables to confirm the effectiveness of the spiritual nursing education programme. Systematic review studies [11, 14] from other countries used various variables such as the Spirituality and Spiritual Care Rating Scale, Spiritual Transcendence Scale, Spiritual Perspective Scale, Spiritual Care Inventory, and Spiritual Care in Practice Survey to confirm the effectiveness of spiritual nursing education. To meet the spiritual needs of nursing students, self-awareness to understand one’s spirituality is crucial [11]. Future studies examining the effectiveness of spiritual nursing education should consider other variables, such as evaluating nurses’ perceptions of spirituality and spiritual nursing [41] and measuring the effect of spiritual nursing performance on nurses [42]. The studies included in this study have limitations in subjective evaluation because all measurements of the effectiveness of spiritual nursing education were measured using self-reported questionnaires. Prior studies from other countries showed that the objectivity of the effect measurement of spiritual nursing education increased after providing spiritual nursing education for nurses [43]. One study objectified the effect of spiritual nursing education by examining the number of spiritual nursing interventions before and after spiritual nursing education [44]. For objective evaluation, evaluation checklists, in which the evaluation criteria are objectively presented not only for self-evaluation, can be used for head nurses or patients. Various other methods can be considered for more objective evaluation by using standardised measurements for the subject’s spiritual nursing intervention skill level, number of executions, performance accuracy, and knowledge level [45]. Therefore, future studies should confirm the effectiveness of spiritual nursing education by using more objective, reliable, and valid measurement methods and tools.

The meta-analysis showed that the spiritual nursing education programme increased spiritual nursing competency, spirituality, spiritual well-being, existential well-being, and spiritual needs. Understanding one’s spirituality helps in understanding the spiritual needs of others by reflecting on one’s own spiritual beliefs in the process of identifying individual spirituality and spiritual needs through self-awareness at the beginning of the spiritual nursing education programme. Spiritual nursing education [14] aim to develop sensitivity to spiritual nursing, clarify the importance of spirituality and spiritual nursing in healthcare, and present a spiritual nursing intervention method. Thus, it can affect the acknowledgement of individual spirituality and the integration of spirituality in clinical practice and communication with patients [14].

However, the quality of studies included may pose the risk of randomisation-related bias because of the minimal number of randomised trials used, with the possibility of the effect estimates of the outcome variables being overestimated when interpreting the results. Moreover, the study had limitations in explaining the effect as only six studies were included in the meta-analysis, most results showed large heterogeneity, and moderation effect analysis was not conducted because less than ten studies were selected.

Nevertheless, this study can be meaningful in a few aspects. We tried to comprehensively and scientifically synthesise individual study results confirming the effectiveness of spiritual nursing education programmes for nurses and nursing students in Korea. In particular, we aim to contribute to the planning of the future directions of spiritual nursing education intervention research by providing the content and teaching methods of programmes. Furthermore, diverse outcome variables were explored and integrated to estimate the significance of the effects of spiritual nursing education programmes.

## Conclusions

This study examined the teaching methods and contents of spiritual nursing education programmes for nurses and nursing students and confirmed their effectiveness. The teaching methods included lectures, discussions, and case presentations, while the contents included self-spiritual awareness, spirituality-related concepts, understanding the spirituality of others, and the spiritual nursing process and application. To confirm the effects of education programmes, we mainly used variables related to spiritual care competency. Spiritual nursing education increased spiritual care competency and individual spirituality. The meta-analysis showed statistically significant effects on spiritual nursing competency, spirituality, spiritual well-being, existential well-being, and spiritual demand, but not on self-esteem. This study’s findings on the characteristics of spiritual nursing education programmes in Korea can help develop and apply programmes for nursing students and nurses. Given the improvement in spiritual nursing competency, more attention and administrative and financial support for spiritual nursing education programmes in schools and hospitals should be provided. To further advance this science, more randomised experimental studies on the effectiveness of spiritual nursing education on clinical nurses is necessary. Furthermore, future studies should examine whether short online training is effective and verify its continued effects through a long-term follow-up study. We also recommend developing and applying spiritual nursing education programmes considering Korean practices, such as spiritual nursing interventions that addresses spiritual needs arising from relationships with others and promoting existential well-being full of meaning and purpose in life.

### Electronic supplementary material

Below is the link to the electronic supplementary material.

Supplementary Material 1

## Data Availability

The supplementary material used for this study can be found in Additional file 1. The datasets used or analysed for the current study are available from the corresponding author on reasonable request.

## Abbreviations

ADDIE: Analysis Design Development Implementation Evaluation
ASSET: Actioning, Spirituality and Spiritual Care Education and Training in nursing
Cochran’s RoB: Cochrane’s Risk of Bias
DBpia: DataBase Periodical Information Academic
ICN: International Council of Nurses
KISS: Korean Studies Information Service System
NDSL: National Discovery for Science Library
PICOSD: Participants, Intervention, Comparison, Outcome, and Study Design
PPT: PowerPoint
PRISMA: Preferred Reporting Items for Systematic Reviews and Meta-Analyses
RCT: Randomised Controlled Trial
RISS: Research Information Sharing Service
RN-BSN: Registered Nurse-Bachelor of Science in Nursing
RoBANS: Risk of Bias Assessment Tool for Non-randomised Studies
SMD: Standardised Mean Difference
