# Supplementary material for: Spiritual nursing education programme for nursing students in Korea: a systematic review and meta-analysis
Source: BMC Nurs. 2024 May 7;23:310. doi: 10.1186/s12912-024-01961-6 (PMC11077795; doi:10.1186/s12912-024-01961-6)
Supplement: Supplementary file 1 — Supplementary Material 1 [file 12912_2024_1961_MOESM1_ESM.pdf]

Additional file 1. List of Studies Included in Systematic review

1. Kim J, Park K. The influences of spiritual care nursing education towards death and dying. J Korean Public Health Nurs. 1999;13:114-27.

2. Chung MJ, Eun Y. Development and effectiveness of a spiritual care education program for nursing students-based on the ASSET model. J Korean Acad Nurs. 2011;41:673-83. doi: 10.4040/jkan.2011.41.5.673.

3. Choi EJ. Effects of the spiritual care education on spiritual well-being and spiritual care competence in nursing student. J Wholist Nurs Sci. 2014;7:143-50.

4. Hong S. Effects of a spiritual care education program based on the action learning on spiritual needs, spiritual well-being and spiritual care competence of nursing students. Jour of KoCon a. 2016;16:285-94. doi: 10.5392/JKCA.2016.16.01.285.

5. Jeong JO, Jo HS, Kim Sh. Effect of the spiritual care module education program for nurses. J Korean Acad Soc Nurs Educ. 2016;22:51-62. doi: 10.5977/jkasne.2016.22.1.51.

6. Yoon MO, Sim JH. The effects of spiritual nursing care education of christian university nursing students. Theol Soc. 2018; 32:221-55.

7. Choi SK, Kim J, Kim S. Development and effectiveness of a spiritual care education program for nurses. J Converg Inf Technol. 2019;9:67-77. doi: 10.22156/CS4SMB.2019.9.9.067.

8. Kim J, Cha NH. Effect of a spiritual care empowerment program on psychological empowerment of nursing students. J East-West Nurs Res. 2019;25:117-27. doi: 10.14370/jewnr.2019.25.2.117.

9. Lim H-J, Park K. The effectiveness of a spiritual education for spiritual care competence reinforcement of nursing students. J Digit Converg. 2021;19:261-74. doi: 10.14400/JDC.2021.19.1.261.
